# An Uncommon Presentation of Renal Angiomyolipoma: A Case Report

**DOI:** 10.7759/cureus.55410

**Published:** 2024-03-02

**Authors:** Asmaa Ahmed, Andrew Takla, Amr Salama, Mohamed S Mohamed, Naila Choudhary

**Affiliations:** 1 Internal Medicine, Rochester General Hospital, Rochester, USA; 2 Cardiology, University of Alabama, Birmingham, USA; 3 Cardiology, Sands-Constellation Heart Institute, Rochester Regional Health, Rochester, USA

**Keywords:** secondary hypertension, renal angiomyolipoma, renal tumor, tuberous sclerosis, uncontrolled hypertension

## Abstract

Renal angiomyolipoma (AML) is a rare benign tumor of the kidney that can occur as a sporadic lesion or a part of tuberous sclerosis. A 77-year-old female patient with a history of hypertension, hyperlipidemia, and an unclear history of left nephrectomy in 1999 presented with progressive shortness of breath and palpitations. Her vital signs showed elevated blood pressure, and the examination was benign and non-focal. A work-up showed multiple lesions in her lungs and right kidney, representing lymphangioleiomyomatosis. The patient was diagnosed with tuberous sclerosis and was followed up by pulmonology and nephrology. She underwent embolization of the renal AML, after which her blood pressure (BP) was more controlled, and she reported feeling well and symptom-free. Renal AML, as a part of tuberous sclerosis, is a rare cause of secondary hypertension. Embolization of AML is effective in controlling BP.

## Introduction

Renal angiomyolipoma (AML) is a rare benign tumor of the kidney. It occurs mostly as a sporadic lesion in the kidney and rarely as a part of tuberous sclerosis presenting with pulmonary lymphangioleiomyomatosis, skin or retinal hamartomas, subependymal giant cell astrocytoma, or heart rhabdomyoma [1]. Presenting symptoms are quite variable, ranging from abdominal pain, hematuria, and hypertension to severe bleeding and shock [1]. Renal disease is the most common cause of death in adults with tuberous sclerosis complex [2]. We are presenting a case of renal AML as a part of the tuberous sclerosis complex presenting as a cause of uncontrolled hypertension.

## Case presentation

A 77-year-old female patient with a history of hypertension, hyperlipidemia, osteoporosis, chronic kidney disease (CKD) with a history of left nephrectomy in 1999, and bronchial asthma presented with progressive shortness of breath. She complained of labored breathing toward the end of any activity associated with palpitation. She did not have chest pain, chest pressure, orthopnea, paroxysmal nocturnal dyspnea, or lower extremity swelling. On physical examination, her blood pressure (BP) was high (160/80), but she had otherwise normal cardiovascular and lung examinations. Given her multiple risk factors, ischemic heart disease was one of the top differentials. Her resting EKG showed sinus rhythm with no ischemic changes. She was started on carvedilol (3.125 mg) twice daily in addition to her daily dose of amlodipine (10 mg). She continued to take aspirin (81 mg) and Lipitor (40 mg) daily, which were previously started by her primary care physician.

**Figure 1:**
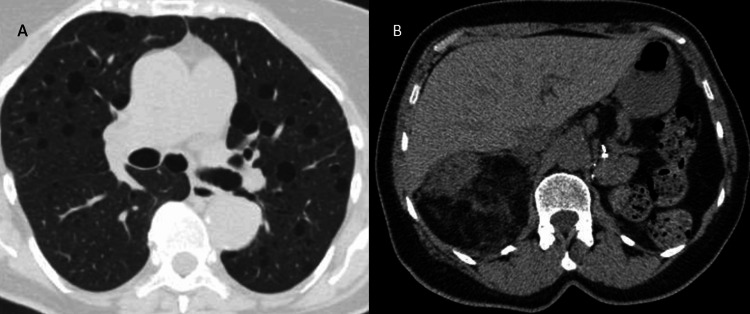
(A) Lung lymphangioleiomyomatosis. (B) Exophytic fat-density masses within the right kidney.

On follow-up, her BP remained high at 173/75, but she never started Coreg. Her echocardiogram was normal, and the stress echocardiogram came back equivocal, so the decision was to do a CT coronary angiogram, which showed mild non-obstructive coronary artery disease (CAD). CT scan also revealed multiple lesions in her lungs and kidney suggestive of lymphangioleiomyomatosis. The largest mass measured 8.2 cm in the right lung in addition to multiple exophytic fat-dense masses within the right kidney; the largest one measured up to 7.6 × 9.0 cm (Figure 1).

**Figure 2:**
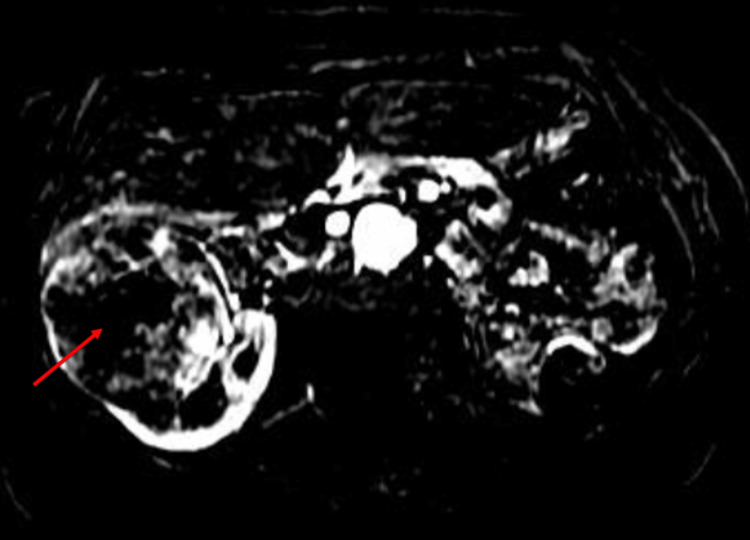
Right renal angiomyolipoma on MRI (red arrow).

Findings were confirmed on MRI (Figure 2).

**Figure 3:**
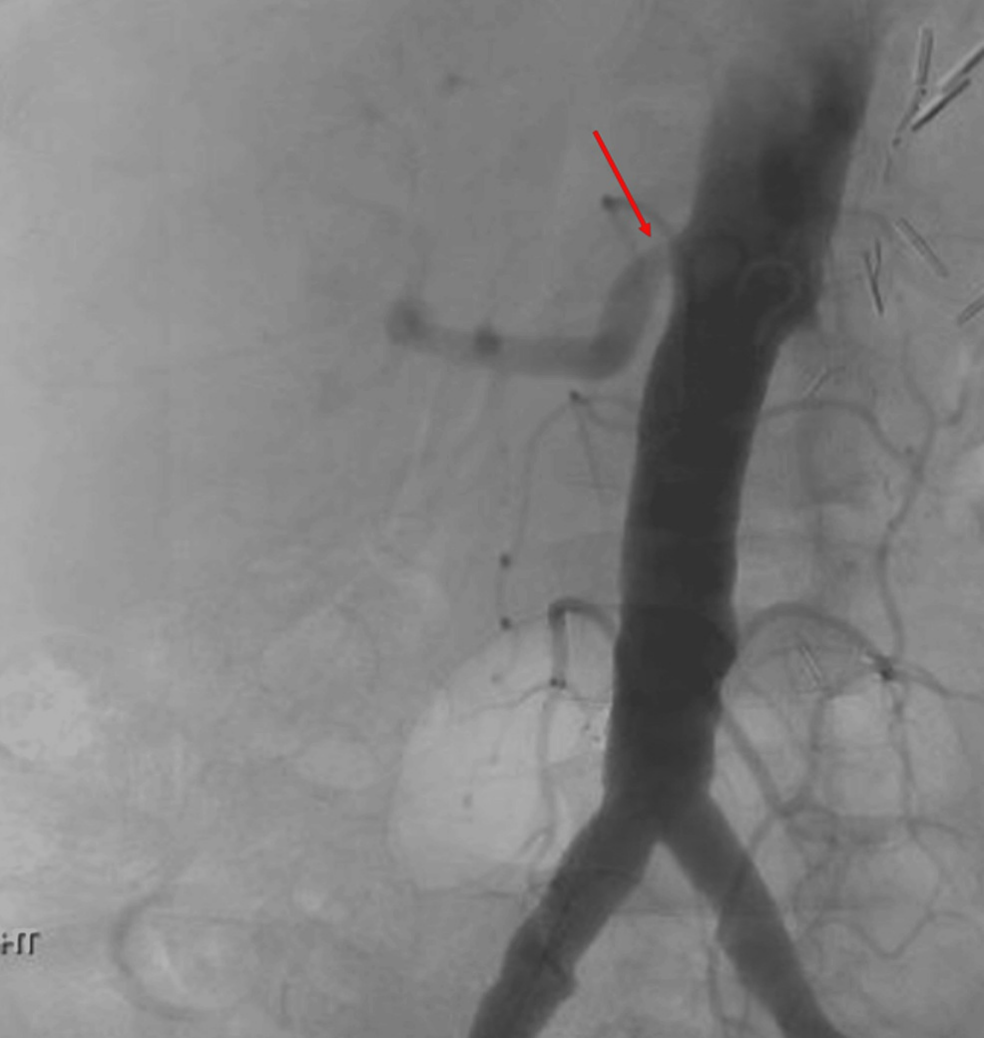
Renal angiogram with focal stenosis (red arrow) at the origin of the right renal artery.

Subsequent renal angiogram showed a hemodynamically significant focal stenotic lesion at the origin of the right renal artery (Figure 3).

She underwent embolization of the renal AML, following which her blood pressure improved, requiring down titration of antihypertensive medications. She also had symptomatic improvement in her shortness of breath with the optimization of asthma treatment. Since her creatinine remained stable at 1.3 and BP was better controlled after AML embolization, the decision was not to do renal artery stenting and continue following up.

## Discussion

The guidelines established by the European Society of Cardiology (ESC)/European Society of Hypertension (ESH) recommend screening for secondary causes of hypertension in select patient populations. These include individuals who develop hypertension at a younger age (<40 years), acute worsening of previously controlled hypertension, severe (grade 3) or drug-resistant hypertension, and the presence of organ damage mediated by hypertension [3]. We are presenting a case of uncontrolled hypertension in a female patient secondary to renal artery stenosis caused by renal AML.

Renal AML is known to be more prevalent in middle-aged women [4]. However, our case presented an older age. Considering the patient's history of prior left nephrectomy for a tumor, we suspect that she has been affected by this condition for over 20 years. Unfortunately, we lack detailed information about her previous presentation. The diagnosis of AML in our case was incidental, occurring during her evaluation for ischemic heart disease, which was prompted by initial symptoms of shortness of breath and uncontrolled blood pressure. Our patient did not exhibit the clinical criteria or the laboratory stigmata associated with other causes of secondary hypertension, such as pheochromocytoma, primary hyperaldosteronism, Cushing syndrome, obstructive sleep apnea, thyroid disease, and parathyroid disease, as mentioned earlier. Reviewing the literature, CT scans and MRI are considered highly reliable diagnostic tools for AML, given their ability to detect the high-fat content typically associated with these tumors [5]. Another noteworthy finding in our case was the presence of a hemodynamically significant stenotic lesion in the right renal artery, which posed additional challenges to the management approach. Typically, management of a stenotic renal artery would involve revascularization, especially in cases of uncontrolled hypertension or declining kidney function [6]. In patients with renal artery stenosis in a solitary kidney and uncontrolled hypertension, management would have typically involved stenting of the renal artery. However, in our patient's situation, the presence of a large AML in the same kidney increased the risk of bleeding associated with stenting, as well as the subsequent use of antiplatelet after stenting. To avoid the risk of spontaneous bleeding and the need for nephrectomy, embolization, and cutting off the blood supply, feeding the AML proved to be the most effective approach as long as blood pressure remains controlled and kidney functions remain stable. Consequently, the multidisciplinary team's decision was to delay the stenting of the renal artery, and the patient was ultimately managed through embolization of the renal artery branch that supplied the AML.

## Conclusions

Renal AML is a rare tumor that might cause secondary hypertension. Embolization of AML can be effective in controlling BP in these cases.
